# Differential Effects of Acute Treatment With Antipsychotic Drugs on Peripheral Catecholamines

**DOI:** 10.3389/fpsyt.2020.617428

**Published:** 2020-12-01

**Authors:** Heidi N. Boyda, Amanzo A. Ho, Lurdes Tse, Ric M. Procyshyn, Jessica W. Y. Yuen, David D. Kim, William G. Honer, Alasdair M. Barr

**Affiliations:** ^1^Department of Anesthesiology, Pharmacology and Therapeutics, Faculty of Medicine, University of British Columbia, Vancouver, BC, Canada; ^2^Department of Psychiatry, Faculty of Medicine, University of British Columbia, Vancouver, BC, Canada

**Keywords:** antipsychotic, norepinephrine, epinephrine, dopamine, catecholamine, rat, metabolic side effects

## Abstract

Antipsychotic drugs represent the most effective treatment for chronic psychotic disorders. The newer second generation drugs offer the advantage of fewer neurological side-effects compared to prior drugs, but many cause serious metabolic side-effects. The underlying physiology of these side-effects is not well-understood, but evidence exists to indicate that the sympathetic nervous system may play an important role. In order to examine this possibility further, we treated separate groups of adult female rats acutely with either the first generation antipsychotic drug haloperidol (0.1 or 1 mg/kg) or the second generation drugs risperidone (0.25 or 2.5 mg/kg), clozapine (2 or 20 mg/kg), olanzapine (3 or 15 mg/kg) or vehicle by intraperitoneal injection. Blood samples were collected prior to drug and then 30, 60, 120, and 180 mins after treatment. Plasma samples were assayed by HPLC-ED for levels of norepinephrine, epinephrine, and dopamine. Results confirmed that all antipsychotics increased peripheral catecholamines, although this was drug and dose dependent. For norepinephrine, haloperidol caused the smallest maximum increase (+158%], followed by risperidone (+793%), olanzapine (+952%) and clozapine (+1,684%). A similar pattern was observed for increases in epinephrine levels by haloperidol (+143%], olanzapine (+529%), risperidone (+617%) then clozapine (+806%). Dopamine levels increased moderately with olanzapine [+174%], risperidone [+271%], and clozapine [+430%]. Interestingly, levels of the catecholamines did not correlate strongly with each other prior to treatment at baseline, but were increasingly correlated after treatment as time proceeded. The results demonstrate antipsychotics can potently regulate peripheral catecholamines, in a manner consistent with their metabolic liability.

## Introduction

The second generation antipsychotic drugs (also known as the “atypical” antipsychotics, to differentiate them from the original first generation, “typical,” antipsychotics) represent the most effective pharmacological treatment for chronic psychotic illnesses, which include the schizophrenia spectrum disorders (1). This class of drugs is also increasingly being used to treat additional psychiatric indications, such as bipolar disorder, mood, and anxiety disorders (2–7). The widespread preference for the use of the second over the first generation antipsychotics is largely driven by the lower incidence of neurological side-effects in the former, which include extrapyramidal symptoms and tardive dyskinesia (8), as well as lower rates of neuroendocrine abnormalities such as hyperprolactinemia (9, 10). The second generation drugs, however, have been linked to their own serious side-effects as well. These most commonly include cardiometabolic side-effects, which significantly increase the risk of developing cardiometabolic disorders such as Type 2 diabetes mellitus (DM) and cardiovascular disease (11–19). Typically, Type 2 DM is preceded by the onset of the metabolic syndrome, which is characterized by weight gain, hypertension, hyperlipidemia, hyperglycemia, glucose intolerance, and insulin resistance (20–22). It is important to note, though, that these metabolic side-effects vary considerably between the different second generation antipsychotics, with some drugs having more severe metabolic effects than others (23, 24).

Currently, the physiological substrates that mediate the metabolic side-effects of the second generation antipsychotic drugs remain incompletely understood. As it is likely that invasive procedures will be required to fully elucidate the biochemical pathways involved, which may not be appropriate for application in humans, there is a key need to develop translational animal models of these drug side-effects. The use of preclinical paradigms offers the additional benefit of helping to disentangle the multifactorial causes of metabolic dysregulation in psychiatric illness, where variables such as diet, exercise, and drug use can all contribute to cardiometabolic dysregulation (25–27). Fortunately, the past 10–15 years has seen significant advances in animal models of the metabolic side-effects of antipsychotic drugs (28), which have demonstrated that many of these compounds cause acute glucose dysregulation and insulin resistance independent of weight gain (29–39).

The biochemical substrates involved are under examination, and important advances have been made. But the present perspective suggests a complex picture, as both central and peripheral mechanisms may be involved (40). It is also possible that multiple biochemical pathways may be affected, upstream, and downstream of each other. One physiological network that spans both central and peripheral systems, and that has been implicated in the metabolic side-effect of antipsychotic drugs in both animal and human studies, is the sympathetic nervous system. Under physiological conditions, an overactive sympathetic nervous system predicts an increase in metabolic abnormalities over time (41). Increased sympathetic activity; as evident by elevated plasma catecholamines, is a consistently reported effect of high metabolic-risk second generation antipsychotic drugs such as clozapine, whereas minimal effects on catecholamine levels are observed in patients treated with lower metabolic-risk drugs (42–44).

While animal studies also suggest a role for peripheral catecholamines (45, 46), a limitation of the literature is the absence of data on the impact of concurrent multiple different antipsychotics, including both first and second generation drugs (to capture the spectrum of metabolic liability), on peripheral catecholamine levels over repeated time points. To address this gap in the literature, we performed a head-to-head comparison of the effects of treatment with the first generation antipsychotic drug haloperidol—with low metabolic liability—against the second generation drugs risperidone, olanzapine and clozapine—which exert increasing metabolic effects. To increase the validity of the study, multiple doses of each drug were used, based on known metabolic effects reported by our laboratory previously (47–51). Animals were unanesthetized and freely moving, handled by a staff well-trained in stress-free phlebotomy, and catecholamine levels were measured with multiple blood draws over a 3-h period. Catecholamines were measured with a sensitive HPLC-ECD assay.

## Materials and Methods

### Animals

One hundred twenty female, adult Sprague-Dawley rats (225–250 g) were obtained from the animal supplier (Charles River, Montreal, QC) and allowed to habituate in the UBC animal colony for at least 1 week before all experiments commenced. Females are used by our laboratory and many other groups because they exhibit more consistent metabolic abnormalities than males following antipsychotic drug treatment (28, 33, 52–54). Animals were housed in groups of 3–4 in large polycarbonate cages and given ad libitum access to food (Purina rat chow) and tap water. All rats were maintained on a 12-h light-dark cycle (lights on at 07:00 h) in a temperature-controlled colony at 22 ± 1°C. Experimental procedures were conducted during the light cycle, and in accordance with the National Institutes of Health Guide for the Care and Use of Laboratory Animals. The University of British Columbia's Animal Care and Use Committee approved all experimental methods.

### Pharmaceutical Agents and Solutions

Doses of antipsychotic drugs were based on previous studies of the metabolic side-effects of antipsychotic drugs which we and others have demonstrated previously. A lower and higher dose were selected for each antipsychotic, spanning a 3–10-fold range. Doses for the present study included risperidone (0.25; 2.5 mg/kg), haloperidol (0.1; 1.0 mg/kg), clozapine (2; 20 mg/kg), and olanzapine (3; 15 mg/kg) [purchased from Toronto Research Chemicals Inc, Toronto, ON, Canada]. Dosing solutions were prepared fresh daily: risperidone, clozapine, and olanzapine were formulated in a vehicle composed of 50% polyethylene glycol 400, 40% distilled water, and 10% ethanol. Haloperidol was formulated in a vehicle of 0.3% tartaric acid. All other chemicals were commercially available and of reagent grade. Each rat received a 1 ml/kg intraperitoneal (IP) injection of the vehicle control or antipsychotic formulation. Animals were all experimentally naïve, and randomized to treatment group.

### Treatment and Plasma Collection

Fasted animals [*n* = 10 per group] were weighed and allowed 1 h to acclimate. We used fasted animals, as this is the condition under which we typically assess the metabolic effects of antipsychotic drugs. Rats were then given a saphenous blood draw (200 μL) prior to drug treatment to obtain a baseline measure of plasma catecholamines. Immediately following, animals were treated with their assigned antipsychotic drug or vehicle. Additional blood samples were then collected at 30, 60, 120, and 180 mins after drug treatment. Blood samples were centrifuged (10,000 RPM, 10 mins, 4°C) and stored at −80°C until analysis.

### Determination of Plasma Catecholamine Concentration

Standard solutions of epinephrine, norepinephrine and dopamine (50–25,600 pg/ml) were prepared by dilution of a stock 1 mg/ml solution with standard diluent (ThermoFisher, Sunnyvale, CA) to generate a standard curve. The internal standard (IS) solution consisted of 20 ng/ml 3,4-dihydroxybenzylamine (DHBA). Samples were prepared by adding 90 μL of 3 M Tris-5% EDTA buffer, 10 μL of DHBA, and 50 μL of plasma or standard to a 0.6 ml centrifuge tubes containing 5.0 mg activated alumina oxide (Sigma Aldrich, St. Louis, MO). Samples were vortexed and placed on a rotary mixer for 10 mins at 4°C, followed by centrifugation and removal of supernatant. Addition of 400 μl of ultra-pure water followed by aspiration was performed three consecutive times, followed by centrifugation. Addition of 50 μl of 0.1 M perchloric acid was added and samples were mixed on the rotary mixer for 10 mins. Samples were vortexed, centrifuged, and the remaining supernatant was injected into the HPLC system.

### HPLC-ECD

Catecholamine levels were analyzed by HPLC coupled to electrochemical detection (ECD). A Shimadzu series HPLC system, including an ESA Coulochem III electrochemical detector, separated analytes on a ESA HR-80 column (80 × 4.6 mm, 3 μm). Mobile phase contained 0.7% sodium phosphate, 3% sodium citrate, 0.02% EDTA, 0.2% diethylamine HCl, 0.1% 1-octanesulfonic acid, 5% acetonitrile, 0.2% dimethylacetamide and water, pH–adjusted to 3.1. A 20 μl injection was loaded at 0.4 ml/min. The ECD system contained an ESA 5020 guard cell (200 mV) and an ESA 5011 analytical cell (E_1_ = −150 mV; E_2_ = 225 mV) for acquisition of epinephrine, norepinephrine, dopamine, and IS plasma levels. Data were processed blind to treatment condition, with chromatogram peaks analyzed by the LCsolution software package.

### Statistical Analysis

Data obtained included plasma levels of norepinephrine, epinephrine, and dopamine. All data were subjected to a repeated-measures analysis of variance (ANOVA), with drug treatment and dose as between group factors, and time as the within-subjects factor. Main effects or interactions were followed up with the LSD *post-hoc* test. Correlations were conducted using the Pearson correlation coefficient (IBM SPSS Statistics for Windows, Version 24.0. Armonk, NY: IBM Corp).

## Results

**Figure 1:**
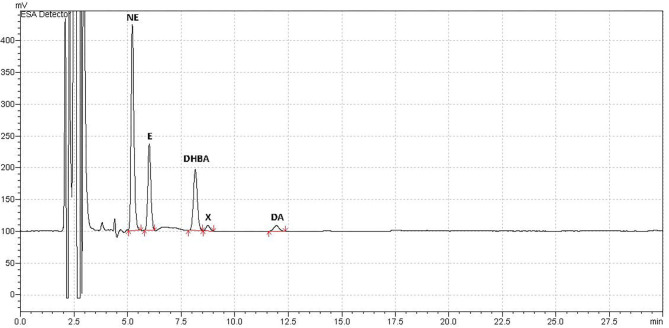
Example Chromatograph. Example of typical chromatograph obtained using HPLC-ED for analysis of plasma catecholamine concentrations in a sample from adult female rats treated with an antipsychotic drug or vehicle. Catecholamine peaks were all well-separated with no peak overlap. Catecholamines measured were norepinephrine (NE), epinephrine (E), and dopamine (DA). The internal standard, added after plasma collection, was 3,4-dihydroxybenzylamine (DHBA). An unidentified peak (“X”) occurred at ~8–8.5 mins, which correlated with catecholamine levels, and may have been a metabolite.

Blood samples were successfully collected for all animals at all time points. Observation of the chromatograms demonstrated that peaks for norepinephrine, epinephrine, and dopamine (as well as the IS) were all well-separated and with no overlap (Figure 1). We also identified an unknown peak at ~8–9 mins (peak “X,” Figure 1), which we confirmed was not serotonin, and may represent a catecholamine metabolite.

### Norepinephrine

**Figure 2:**
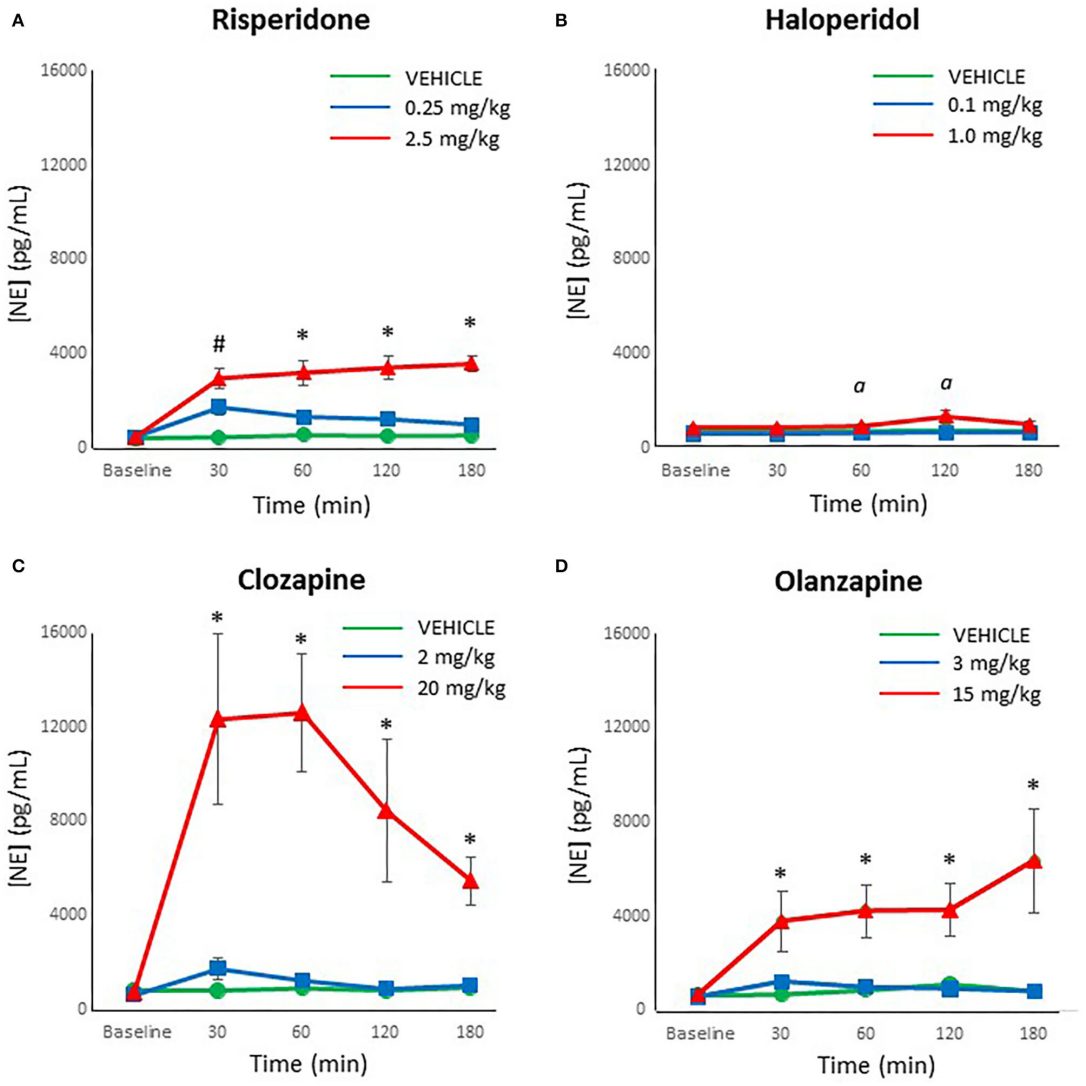
Acute changes in plasma norepinephrine (NE) concentrations (pg/ml) after antipsychotic drug treatment in adult female rats. Animals (*n* = 10 per group) were given a single IP injection of **(A)** risperidone (0.25 or 2.5 mg/kg) or **(B)** haloperidol (0.1 or 1.0 mg/kg) or **(C)** clozapine (2 or 20 mg/kg) or **(D)** olanzapine (3 or 15 mg/kg) or vehicle. Values are expressed in group means ± SEM. Plasma samples were collected immediately prior to drug treatment (Baseline) and at 30, 60, 120, and 180 mins after treatment. ^#^Statistically significant difference between both drug treated groups and vehicle, *p* < 0.05; ^*^Statistically significant difference between high dose group and both other groups, *p* < 0.05; α Statistically significant difference between high dose group and vehicle, *p* < 0.05.

When reviewing the data on norepinephrine levels, an analysis for outliers determined that two samples were strong outliers based on SPSS criteria (>3 × the interquartile range) and so were excluded from analysis and replaced with next observation carried backward (55). Analyzing each drug individually, risperidone demonstrated a significant effect of dose [*F*_(2,27)_ = 31.44, *p* < 0.001], time [*F*_(4,108)_ = 29.23, *p* < 0.001] and a dose × time interaction [*F*_(8,108)_ = 13.71, *p* < 0.001]. *Post-hoc* analysis revealed that both doses of risperidone caused a significant increase in norepinephrine levels by 30 mins compared to controls, but by 60 mins the lower dose of risperidone did not differ from controls, for the duration of the test (Figure 2A). By contrast, norepinephrine levels remained significantly elevated at all time points after treatment with the higher dose of risperidone compared to both the low dose of risperidone and controls.

For haloperidol, the ANOVA indicated a significant effect of dose [*F*_(2,27)_ = 7.31, *p* < 0.005], time [*F*_(4,108)_ = 5.80, *p* < 0.001] and a dose × time interaction [*F*_(8,108)_ = 3.01, *p* < 0.005]. *Post-hoc* analysis confirmed that levels of norepinephrine with the low dose of haloperidol did not differ at any time point from controls (Figure 2B). In contrast, the high dose of haloperidol was associated with a trend for increased norepinephrine levels compared to controls at 30 mins after treatment (*p* = 0.06), and then significantly higher between 60 and 120 mins (*p* < 0.05), but no longer at 180 mins (*p* = 0.07).

With clozapine, the ANOVA indicated a significant effect of dose [*F*_(2,27)_ = 18.60, *p* < 0.001], time [*F*_(4,108)_ = 6.82, *p* < 0.001] and a dose × time interaction [*F*_(8,108)_ = 5.56, *p* < 0.001]. *Post-hoc* analysis demonstrated that levels of norepinephrine with the low dose of clozapine did not differ at any time point from controls (Figure 2C). However, the high dose of clozapine evinced a large, highly significant increase in norepinephrine levels compared to both the controls and the low dose of clozapine at all time points after treatment (*p* < 0.001).

For olanzapine, analysis of the data by the ANOVA revealed a significant effect of dose [*F*_(2,27)_ = 11.07, *p* < 0.001], time [*F*_(4,108)_ = 4.00, *p* = 0.005] and a dose × time interaction [*F*_(8,108)_ = 3.08, *p* < 0.005]. Similarly to clozapine, the *post-hoc* analysis indicated that levels of norepinephrine with the low dose of olanzapine did not differ at any time point from controls (Figure 2D). In contrast, the high dose of olanzapine caused a large, significant increase in norepinephrine levels compared to both the controls and the low dose of clozapine at all time points after treatment (*p* < 0.01).

### Epinephrine

**Figure 3:**
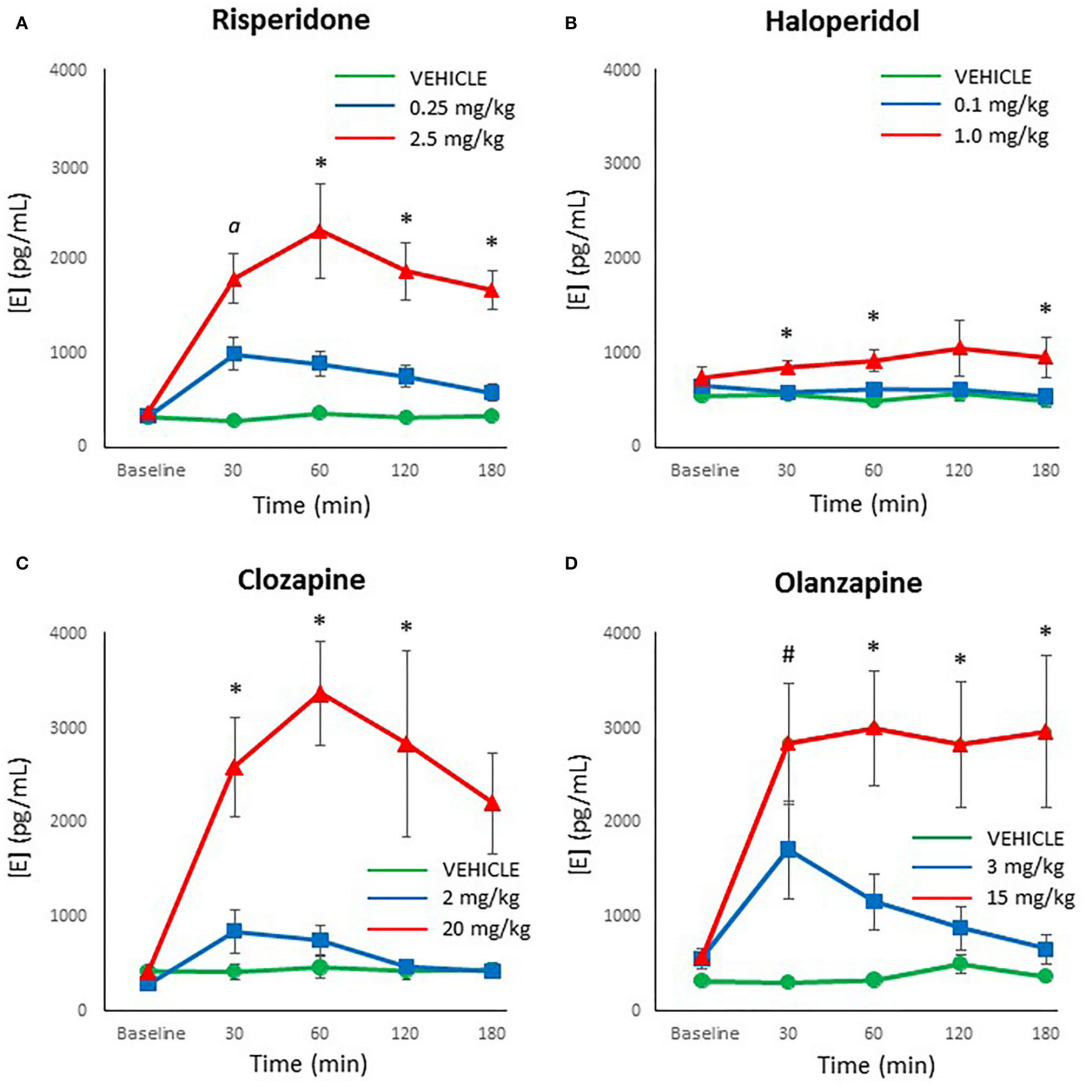
Acute changes in plasma epinephrine (E) concentrations (pg/ml) after antipsychotic drug treatment in adult female rats. Animals (*n* = 10 per group) were given a single IP injection of **(A)** risperidone (0.25 or 2.5 mg/kg) or **(B)** haloperidol (0.1 or 1.0 mg/kg) or **(C)** clozapine (2 or 20 mg/kg) or **(D)** olanzapine (3 or 15 mg/kg) or vehicle. Values are expressed in group means ± SEM. Plasma samples were collected immediately prior to drug treatment (Baseline) and at 30, 60, 120, and 180 mins after treatment. α Statistically significant difference between all three groups, *p* < 0.05; ^*^Statistically significant difference between high dose group and both other groups, *p* < 0.05; ^#^Statistically significant difference between both drug treated groups and vehicle, *p* < 0.05.

Epinephrine levels were reliably measured with the HPLC-ED, with no samples below the limit of detection. Looking at each drug individually, the ANOVA noted that risperidone demonstrated a significant effect of dose [*F*_(2,27)_ = 20.17, *p* < 0.001], time [*F*_(4,108)_ = 17.11, *p* < 0.001] and a dose × time interaction [*F*_(8,108)_ = 7.97, *p* < 0.001]. The *post-hoc* analysis revealed that both doses of risperidone caused a significant increase in epinephrine levels by 30 mins compared to controls (*p* ≤ 0.01), but by 60 mins the lower dose of risperidone did not differ significantly from controls, for the duration of the test (Figure 3A). By contrast, epinephrine levels remained significantly elevated at all time points after treatment with the higher dose of risperidone compared to both the low dose of risperidone and controls (*p* ≤ 0.005).

Analysis of the results with haloperidol by ANOVA indicated a significant effect of dose [*F*_(2,27)_ = 4.05, *p* < 0.05], but no main effect of time or dose × time interaction. *Post-hoc* analysis of the dose effect showed that the low dose of haloperidol and controls did not differ at any time point (Figure 3B), but the high dose of haloperidol caused a significant increase in epinephrine levels compared to both other groups at 30, 60, and 180 mins (*p* < 0.05).

For clozapine, ANOVA indicated a significant effect of dose [*F*_(2,27)_ = 14.15, *p* < 0.001], time [*F*_(4,108)_ = 8.14, *p* < 0.001] and a dose × time interaction [*F*_(8,108)_ = 5.27, *p* < 0.001]. The *post-hoc* analysis confirmed that showed that the low dose of clozapine and controls did not differ at any time point in epinephrine levels (Figure 3C), but the high dose of clozapine had higher epinephrine levels than both other groups at all time points after drug treatment (*p* < 0.01).

With olanzapine, the analysis showed significant main effects of dose [*F*_(2,27)_ = 14.05, *p* < 0.001], time [*F*_(4,108)_ = 6.51, *p* < 0.001] and a dose × time interaction [*F*_(8,108)_ = 3.77, *p* = 0.001]. Follow-up *post-hoc* tests revealed that both doses of olanzapine caused a significant increase in epinephrine levels by 30 mins compared to controls (*p* ≤ 0.01) (Figure 3D), but by 60 mins the lower dose of olanzapine did not differ from controls, for the duration of the test. Epinephrine levels with the higher dose of olanzapine remained significantly higher than both other groups from 60 through to 180 mins (*p* < 0.005).

### Dopamine

**Figure 4:**
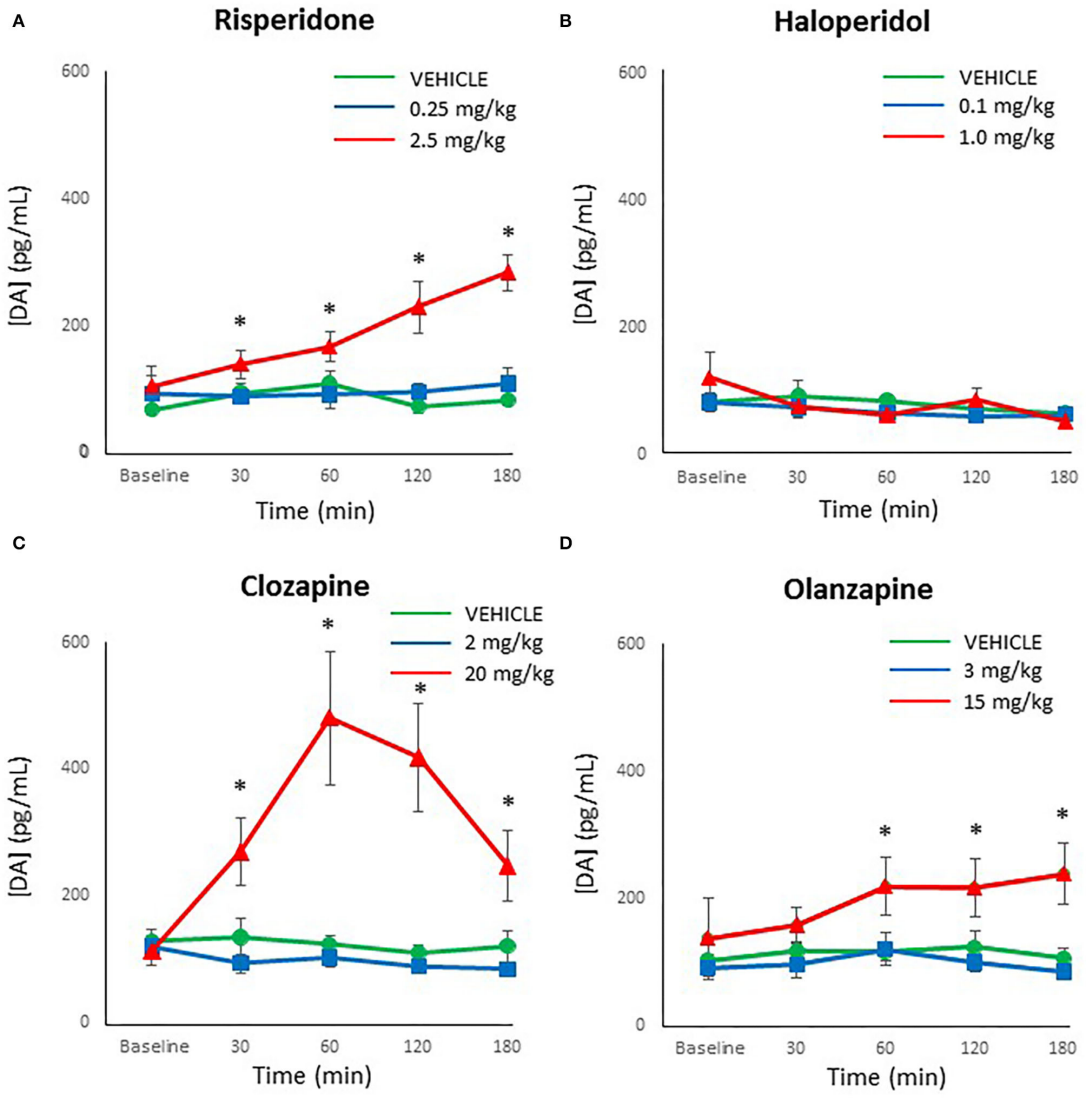
Acute changes in plasma dopamine (DA) concentrations (pg/ml) after antipsychotic drug treatment in adult female rats. Animals (*n* = 10 per group) were given a single IP injection of **(A)** risperidone (0.25 or 2.5 mg/kg) or **(B)** haloperidol (0.1 or 1.0 mg/kg) or **(C)** clozapine (2 or 20 mg/kg) or **(D)** olanzapine (3 or 15 mg/kg) or vehicle. Values are expressed in group means ± SEM. Plasma samples were collected immediately prior to drug treatment (Baseline) and at 30, 60, 120, and 180 mins after treatment. ^*^Statistically significant difference between high dose group and both other groups, *p* < 0.05.

Although dopamine levels were substantially lower than both norepinephrine and epinephrine, dopamine was reliably detected and above the limit of detection in all processed plasma samples. An analysis for outliers determined that three samples were strong outliers (> 3 × the interquartile range) and so were excluded from analysis and replaced with next observation carried backward (55). ANOVA indicated a main effect of dose of risperidone on dopamine levels [*F*_(2,27)_ = 19.60, *p* < 0.001], time [*F*_(4,108)_ = 4.93, *p* = 0.001], and a dose × time interaction [*F*_(8,108)_ = 3.97, *p* = 0.001]. Follow-up *post-hoc* tests showed that while low dose risperidone and vehicle-treated rats did not differ at any time point in dopamine levels, the levels were significantly higher in the high dose group compared to the low dose group at 30 and 60 mins (*p* < 0.05) (Figure 4A), with a strong trend vs. controls (*p* = 0.057 and 0.072, respectively). By 120–180 mins, the high dose exhibited higher levels of dopamine than both other groups (*p* ≤ 0.001).

For haloperidol, there was a main effect of time [*F*_(4,108)_ = 2.51, *p* < 0.05] but no effect of dose or dose × time interaction. This time effect reflected a gradual reduction of dopamine levels in all groups over time (Figure 4B).

With clozapine, the ANOVA indicated a main effect of dose [*F*_(2,27)_ = 11.48, *p* < 0.001], time [*F*_(4,108)_ = 6.79, *p* < 0.001] and a dose × time interaction [*F*_(8,108)_ = 8.44, *p* < 0.001]. Follow-up *post-hoc* tests revealed that low dose clozapine and controls did not differ at any time point (Figure 4C), but high dose clozapine demonstrated higher dopamine levels than both other groups at all timepoints after treatment (*p* < 0.05).

Analysis of olanzapine data by ANOVA indicated a main effect of dose [*F*_(2,27)_ = 7.50, *p* < 0.001] but no effect of time or dose × time interaction. Dopamine levels were higher in the high dose olanzapine group than both other groups from 60–180 mins (*p* < 0.05) (Figure 4D).

### Comparison Between Drugs

As the low doses of antipsychotics did not strongly affect catecholamine levels, we limited our head-to-head comparison of different drugs to the high doses. Baseline norepinephrine levels were included as a covariate for post-treatment norepinephrine levels. The ANOVA indicated a main effect of drug [*F*_(3,36)_ = 10.02, *p* < 0.001], no effect of time, and a drug × time interaction [*F*_(9,108)_ = 2.75, *p* < 0.01]. Follow-up analysis revealed that norepinephrine levels were higher in the clozapine group than all others from 30 to 60 mins (*p* < 0.05), and higher than haloperidol from 120 to 180 mins. Olanzapine had marginally higher levels than haloperidol at 180 mins (*p* = 0.055). For epinephrine, ANOVA indicated a main effect of drug [*F*_(3,36)_ = 4.65, *p* < 0.01] and no effect of time or drug × time interaction. *Post-hoc* analyses demonstrated that risperidone, clozapine, and risperidone had higher epinephrine levels than haloperidol at all points after drug treatment. With dopamine, there were main effects of drug [*F*_(3,36)_ = 8.34, *p* < 0.001], time [*F*_(3,108)_ = 6.12, *p* = 0.001], and a dose × time interaction [*F*_(9,108)_ = 5.54, *p* < 0.001], whereby clozapine-treated animals had higher dopamine levels than all other groups from 30 to 120 mins, and all groups had higher levels than haloperidol at 180 mins.

### Relationship Between Catecholamines

As an exploratory analysis, we examined the relationship between the three catecholamines at each time point (all treatment groups were combined). At pre-treatment baseline, there was a moderate correlation between norepinephrine and the other two catecholamines (*r* = 0.31–0.36, *p* < 0.001) while epinephrine and dopamine were not correlated (*r* = 0.02, NS). Following drug treatment at 30 mins, all catecholamine levels showed a large increase in their correlation with each other (*r* = 0.50–0.73, *p* < 0.001), which increased further at 60 mins (*r* = 0.67–0.88, *p* < 0.001), at 120 mins (*r* = 0.59–0.79, *p* < 0.001) and at 180 mins (*r* = 0.70–0.91, *p* < 0.001).

## Discussion

In the present study, we compared treatment in freely moving rats with multiple doses of four different antipsychotic drugs, over a 180-mins period, on levels of peripheral catecholamines. The drugs included the first generation antipsychotic drug haloperidol, as well as the second generation drugs risperidone, olanzapine and clozapine. Metabolic abnormalities in patients are most common with the latter two drugs (21, 56), although can occur with all antipsychotic medications, including first generation drugs (57). The key results of the study were that all antipsychotics caused an increase in plasma catecholamine levels. For norepinephrine, this varied considerably by drug, and maximal increases relative to baseline for risperidone were [low dose: +382%; high dose: +793%], haloperidol [low dose: no change; high dose: +158%], clozapine [low dose: +273%; high dose: +1,684%] and olanzapine [low dose: +212%; high dose: +952%]. A similar pattern was observed with epinephrine, where maximal changes relative to baseline for risperidone were [low dose: +299%; high dose: +617%], haloperidol [low dose: no change; high dose: +143%], clozapine [low dose: +293%; high dose: +806%] and olanzapine [low dose: +307%; high dose: +529%]. Increases in peripheral dopamine were smaller, showing no change with the low dose of each drug, and only moderate increases relative to baseline with the high dose for risperidone [+271%], haloperidol [no change], clozapine [+430%] and olanzapine [+174%]. Interestingly, levels of the catecholamines did not correlate strongly with each other prior to treatment at baseline, but were increasingly correlated after treatment as time proceeded.

Overall, these findings clearly demonstrate that antipsychotic drugs can increase peripheral plasma catecholamines in a dose- and drug-dependent manner. The smallest increases in catecholamines were caused by haloperidol, followed by risperidone/olanzapine, and largest by clozapine. These findings are in general agreement with the limited literature on the effects of antipsychotic drugs on peripheral catecholamine levels in animals and humans. An earlier study observed that intravenous treatment of rats with the first generation antipsychotic chlorpromazine resulted in a dose-dependent elevation of both norepinephrine and epinephrine to levels comparable to those observed in the present study with the second generation drugs (58). Of interest, chlorpromazine has been characterized as the first generation drug most likely to cause metabolic syndrome (59). More recently, a pair of studies by the same research group noted that intravenous olanzapine and clozapine both caused significant increases in plasma epinephrine, to a comparable degree to the present study (46, 60); norepinephrine and dopamine were not measured in their studies. Indirect evidence for elevated peripheral catecholamines following treatment with clozapine and chlorpromazine was provided by Savoy et al. (45), who demonstrated that the hyperglycemia caused by these two drugs could be decreased by treatment with the ganglionic blocker hexamethonium, which presumably worked by preventing the release of peripheral norepinephrine and epinephrine. Clinical studies of patients treated with antipsychotic drugs have also reliably observed increases in peripheral catecholamines with higher metabolic liability drugs, such as clozapine (42, 44, 61, 62). For example, plasma norepinephrine levels were ~three times higher in patients treated with clozapine than in controls or patients treated with the first generation antipsychotics haloperidol and fluphenazine (42, 44). We are not aware of any studies that have measured the effects of antipsychotic drugs on peripheral dopamine, which may be due to the greater sensitivity of the assay needed.

While the link between elevated peripheral catecholamines and the metabolic effects of antipsychotics was not determined in this study (as we did not measure metabolic indices), norepinephrine and epinephrine are well-established as two of the most potent hormones involved in the regulation of blood glucose levels. They tightly control insulin release, glucagon secretion, hepatic gluconeogenesis, and glycogenolysis (41, 63), and elevated levels of these catecholamines are related to metabolic dysregulation and metabolic syndrome (64). As such, the observed increased in catecholamines provide a possible pathway to hyperglycemia that requires further study. However, the catecholamines may represent only part of the story. The present doses of drugs were based on prior studies by our laboratory, that observed effects on glucose intolerance and insulin resistance (32, 34, 47, 48). These doses were selected to produce metabolic effects comparable in magnitude to metabolic effects observed in humans treated with the same drugs (65, 66). Some of those doses, such as the high dose (1 mg/kg) of haloperidol and the low dose of clozapine (2 mg/kg), previously exerted pronounced effects in the glucose tolerance test (47), yet those same doses currently only produced very modest increases in catecholamines. Whether these are sufficient to produce metabolic dysregulation will require additional experimentation. The role of peripheral dopamine also requires further evaluation; the hormone has diverse functions peripherally, and is mainly co-released from sympathetic nerve fibers with norepinephrine (67). There is considerable evidence to show that peripheral dopamine regulates body weight and glucose homeostasis via insulin release (68, 69), and so may represent an unexplored contributor to the metabolic effects of antipsychotics.

Importantly, the observed effects were all determined in fasted animals, as this is the condition under which we and many other groups typically assess the metabolic effects of antipsychotic drugs (28). Fasting is associated with decreased sympathetic activity (70), whereas carbohydrate and fat feeding causes increased sympathetic activity (71, 72). The present use of fasting animals therefore likely maximized the capacity to observe increases in catecholamines and reduced pre-treatment variability in catecholamine levels between animals.

The mechanism by which antipsychotics increase peripheral catecholamines remains unknown. One potential substrate is the α_2_-adrenoceptor, which acts as an autoreceptor to decrease catecholamine release from nerve terminals (41, 73). Many antipsychotics have a strong affinity for the α_2_-adrenoceptor, and clozapine (which caused the greatest increase of peripheral catecholamines) has the highest α_2_ receptor load (74). However, high α_2_ receptor loads are also associated with antipsychotics with minimal metabolic liability, such as asenapine (32, 74), suggesting that this may not be the main mechanism. We would posit that antipsychotic drugs may be acting centrally in brain regions such as the locus coeruleus (75) or the lateral and paraventricular nuclei of the hypothalamus (76), which serve to activate the sympathetic nervous system and have been implicated in the metabolic effects of antipsychotics (77). The possibility certainly remains, though, that antipsychotics could be exerting peripheral effects on the sympathetic nervous system, such as through their affinity for the monoamine transporters (78).

The current study has a number of limitations. The first of these is that antipsychotic drugs were only administered to female rats, and not to males. The basis for this decision was that female rats typically produce more reliable and robust metabolic effects than do male rats when treated with antipsychotic drugs (28, 52, 53), and so better model the human condition. However, the physiological basis for these sex differences remains incompletely understood, and could feasibly be related to sex differences in catecholamine release (79). This straightforward experiment therefore represents a logical next step in following up the present results. Another limitation of the present study is the acute nature of drug treatment, as animals were only given a single injection of the drug. We would predict that increases in catecholamine levels would not change with repeated treatment, as we have found the metabolic effects of antipsychotics to be stable in rats over time in longitudinal studies (34, 50), and reports of elevated catecholamines in antipsychotic-treated patients include subjects who have also been administered the drugs chronically (42, 44, 61, 62). Nevertheless, the current effects should be determined in rats treated over an extended period with antipsychotic drugs to confirm that the findings remain consistent, and thus better model clinical populations. It would also be informative to assess plasma antipsychotic drug levels in animals treated with the current doses of drugs. Finally, the present study did not concurrently measure both catecholamine release and metabolic dysregulation to confirm, at an individual level, the link between elevated catecholamines and glucose dysregulation. While this would have been informative, there was a conscious decision to progress in a step-by-step manner, which partly comes from a legitimate concern that additional blood draws, injections, and a glucose challenge could significantly have affected stress levels and thus modified catecholamine release. However, future studies will clearly be needed to determine whether these increased catecholamines are causal to metabolic dysregulation using well-designed experimental protocols.

In conclusion, the present results provide strong evidence that antipsychotic drugs exert potent stimulatory effects on peripheral catecholamines, which may be relevant to drug metabolic side-effects. The observation that even the first generation antipsychotic drug haloperidol can significantly increase catecholamine levels with a sufficient dose is consistent with the clinical observation that many first generation drugs are not metabolically neutral and may be associated with metabolic dysregulation (22, 80), albeit milder than most second generation drugs. To our knowledge, the present preclinical study is the first to compare multiple antipsychotics head-to-head, and is also the first to report effects on all three peripheral catecholamine levels. Time-series data collected from unrestrained, unanesthetized animals by a team experienced in stress-free phlebotomy increase the validity of the data. However, many questions remain regarding the direct relationship of these catecholamines to metabolic dysregulation, as well as the physiological pathways involved in catecholamine release, and so significant further research in required on this topic, including assessment of complementary indices of sympathetic activity.

## Data Availability Statement

The raw data supporting the conclusions of this article will be made available by the authors, without undue reservation.

## Ethics Statement

The animal study was reviewed and approved by UBC Animal Care Committee.

## Author Contributions

HB, AH, and LT collected and processed data. AB designed and supervised the study. All authors contributed to the writing and final draft of this manuscript and approve it for publication.

## Conflict of Interest

WH has received consulting fees or sat on paid advisory boards for Otsuka/Lundbeck, Newron, AlphaSights, Guidepoint, Translational Life Sciences and holds shares in Translational Life Sciences and Eli Lilly. He was additionally supported by the Jack Bell Chair in Schizophrenia. RP has been a member of the following advisory boards in the past 3 years: Janssen, Lundbeck, and Otsuka; a member of the following speaker's bureaus in the past 3 years: Janssen, Lundbeck, and Otsuka; and received grants from the Canadian Institutes of Health Research. The remaining authors declare that the research was conducted in the absence of any commercial or financial relationships that could be construed as a potential conflict of interest.
